# Assessing the Prevalence of Risk Factors for Neglected Tropical Diseases in Brazos County, Texas

**DOI:** 10.1371/currents.outbreaks.93540c6c8c7831670591b0264479269c

**Published:** 2017-10-04

**Authors:** Jennifer Horney, Daniel Goldberg, Tracy Hammond, Kahler Stone, Seth Smitherman

**Affiliations:** Department of Epidemiology and Biostatistics, Texas A&M University, College Station, Texas, United States; Department of Geography, Texas A&M University, College Station, Texas, United States; Department of Computer Science and Engineering, Texas A&M University, College Station, Texas, United States; Department of Epidemiology and Biostatistics, School of Public Health, Texas A&M University, College Station, Texas, United States; College of Veterinary Medicine and Biomedical Sciences, Texas A&M University, College Station, Texas, United States

## Abstract

**Introduction:**

Although more than one billion people live at risk of neglected tropical diseases (NTDs) in areas of Asia, sub-Saharan Africa, and Latin America, the degree to which they burden countries like the U.S. is unclear. Even though many NTDs such as dengue, leishmaniasis, and Chagas disease are typically not endemic to the U.S., the possibility of their emergence is noteworthy, especially in states like Texas with high levels of poverty, large immigrant populations, geographic proximity to endemic areas, and a climate amenable to the vectors for these diseases. Despite the health threat that emerging NTDs may pose, little is known about the prevalence of risk factors for NTDs in the U.S.

**Methods:**

We tested the Community Assessment for Public Health Emergency Response (CASPER) method to assess the prevalence of risk factors for NTDs in Brazos County, Texas.

Results: We found relatively low prevalence of risk factors related to travel (5.2% of respondents visited an endemic area in the previous 3 months); however, few respondents reported adherence to mosquito prevention, such as wearing long sleeves and long pants (14.1%, 95% CI: 13.9,14.4) and repellant containing DEET (13.5%, 95% CI: 13.2,13.7). Between 5.4% and 35.8% of respondents had a visible container (e.g., pet water dishes, flower pots, bird baths) that could support mosquito breeding.

**Discussion:**

CASPER findings present public health authorities with potential avenues for implementing health education and other interventions aimed at reducing exposure to risk factors for NTDs among Texas residents.

## Introduction

Neglected tropical diseases (NTDs) are communicable diseases common in subtropical and tropical countries that cause a substantial disease burden due to high morbidity rates. Across the world, over 1 billion people live in at-risk areas 1. Although NTDs are most commonly found in developing countries, developed countries also report NTDs, most frequently associated with travel to an endemic region. In the U.S., the State of Texas could have a high potential public health burden associated with NTDs due to its high poverty rate, large immigrant population, geographic proximity to endemic areas, and a climate amenable to vector life cycles, where applicable 2.

Over the past 10 years, Texas has seen the emergence of several vector-borne viral and parasitic diseases; of these: three are of major public health concern. Dengue fever is caused by five virus serotypes that are spread by mosquitoes. Mosquitoes are infected when they bite a person with dengue virus in their blood and transmit virus after an incubation period to other people via a mosquito bite. There is evidence to suggest that dengue fever has reestablished itself as endemic to the U.S.-Mexico border region. A 2005 study of Brownsville, Texas, residents found a seroprevalence of 39% and an incidence of 4% 3. In 2013, the Centers for Disease Control (CDC) reported an outbreak of 53 confirmed cases of dengue in Texas’s southernmost counties 4.

Leishmaniasis is a parasitic disease caused by *Leishmania mexicana*, the vectors of which are sandflies of the genus *Lutzomyia*. These vectors have an increasingly wide geographic range in the U.S. 5. Unique risk factors for leishmaniasis include: rural residence, frequent interaction with nature, and wildlife hosts residing in or near the home. Reservoirs include wood rats, cotton rats, opossums, and armadillos 6. Suspected cases of locally-acquired leishmaniasis have been reported in southern Texas sporadically for decades 6. However, leishmaniasis seems to have been increasing in range since 2000, with ecological models indicating presence of rodent reservoir species as far as the Canadian border due to climate change 7^,^^8^^,^^9^. Cases or clusters of cases of cutaneous leishmaniasis have been identified in Dallas and Waco, Texas, and McCurtain County, Oklahoma, areas that had never previously reported autochthonous cases 10^,^^11^.

Chagas disease is a parasitic infection caused by *Trypanosoma cruzi* and transmitted by kissing bugs of the genus *Triatoma*. Although the highest density and diversity of kissing bugs species can be found in the Southwestern U.S., kissing bugs species have been reported in at least twenty-eight states 12. Risk factors for Chagas include poor housing quality (e.g., cracks, gaps, poor roofing) and the presence of infected dogs in or around the home or raising chickens near the home, which increases vector density by providing a stable food source 13. Previous data also suggest that stray dogs are frequently carriers; therefore, large numbers of stray dogs in a community should also be considered a risk factor 14. Chagas disease is known to regularly infect both humans and animals in Texas. There are an estimated 300,000 cases of Chagas disease in the U.S., and a recent study estimated a 9% seroprevalence of Chagas in shelter dogs across Texas 12^,^^14^. A mail-in submission program for kissing bugs showed a 63.3% prevalence of Chagas disease in submitted bugs 15.

Soil transmitted helminthes include ascariasis, hookworm, trichuriasis, taeniasis, cysticercosis, echinococcosis, paragonimiasis, and fascioliasis. These diseases, caused by various types of parasitic worms, are transmitted by the ingestion of fully developed eggs, commonly found in soil, water, or produce contaminated with infected feces. Recent estimates suggest that most Latin Americans live at-risk of ascariasis, trichuriasis, and hookworm 16. Although infection rates are still low in the U.S., infections do occur in areas with the proper conditions. For example, a 2004 cross border seroprevalence study of El Paso and Ciudad Juarez found a 3.3% prevalence of Taenia spp., with most Taenia infections occurring on the El Paso side of the border 17. Risk factors include unsanitary conditions, such as lack of proper sewage disposal 18, drinking unsanitary water 19, not washing produce before consumption, and consumption of undercooked pork and seafood. Some of these diseases also involve animal reservoirs; for example, cysticercosis is associated with pig husbandry and cystic echinococcosis commonly occurs in sheep ranchers.

Like most states, Texas has a passive surveillance system for infectious diseases, and cases of most NTDs are already reportable within one week per the state's communicable disease reporting laws 20. However, even with such a surveillance program, NTDs are most likely being underreported. Apart from a few communicable diseases of concern like tuberculosis, infectious diseases are generally reported to public health authorities less than half the time 21. In the case of dengue and other arboviral diseases, symptoms are usually nonspecific and frequently misdiagnosed, especially in the case of mild infections. Because most NTDs are not yet endemic in the U.S., U.S.-based health care personnel are less familiar with these diseases and may not consider them in their differential diagnoses. Additionally, some vector-borne disease infections may result in no symptoms, allowing a disease to persist in human, animal, or vector populations and making them unlikely to be detected through passive surveillance. With limited resources, it may be difficult for health departments to have the capacity necessary for all types of human and vector surveillance, while the sporadic nature of outbreaks makes it difficult to collect baseline data.

While passive surveillance and the monitoring of vector populations are useful in establishing priorities and guiding policy decisions, this alone cannot paint a complete picture of NTDs in Texas. In response to the potential threat posed by NTDs, Texas House Bill 2055 established a sentinel surveillance program in Texas focusing on NTDs of interest in the state, particularly Chagas, leishmaniasis, dengue, ascariasis, hookworm, trichuriasis, taeniasis/cysticercosis, echinococcosis, paragonimiasis, and fascioliasis 22. In order to assess the potential future burden of these diseases, systematic collection of risk factor prevalence data is needed. The Community Assessment for Public Health Emergency Response (CASPER) is a method that could potentially be used to accomplish these goals. CASPER is an epidemiologic method designed to provide household-level information about an affected community’s needs quickly and at a relatively low cost. Although initially adapted by the Centers for Disease Control and Prevention (CDC) from the World Health Organization’s Expanded Program on Immunization for use post-disaster, CASPER has been utilized to assess household preparedness, underlying vulnerabilities, and community perceptions regarding public health emergencies such as H1N1 novel Influenza A and other hazards 23^,^^24^. Recently, CASPER was utilized by the Austin-Travis Public Health Department and Williamson Counties and Cities Health District to assess their community’s knowledge of Zika virus and mosquito bite prevention 25^,^^26^. While this CASPER was novel in that it was focused on aspects of Zika virus, the utility of CASPER to systematically assess the prevalence of risk factors for infectious diseases has not yet been documented. We hypothesize that CASPER can provide state and local health departments with relatively quick and cost-effective access to data that can be used to make informed decisions about the allocation of resources to address risk factors for NTDs.

## Methods

**Ethics Statement**

The survey and associated consent materials were reviewed and approved by the Texas A&M University Institutional Review Board (IRB 2016-0495D). Selected households were approached by an interview team and a qualifying respondent for the household who was 18 years old or older gave written informed consent prior to the start of the interview.

**Study Location**

Brazos County, Texas (Fig 1) is located in central east Texas and is the location of Texas A&M University, a public university with an enrollment of nearly 60,000 students located on over 5,200 acres of land. In 2015, Brazos County had an estimated population of 215,000, which was 10.4% greater than its 2010 population 27. It is home to a single metropolitan statistical area known as Bryan-College Station as well as a surrounding rural area of about 600 square miles 28.

**Data Sources**

A population-based sample in Brazos County, Texas, (Fig 1) was selected using the Collect SMART Survey Management and Response Tool, a suite of software developed to help users design and implement a CASPER 29. Thirty census blocks were selected probability proportionate to population and seven households from each cluster were sequentially selected from a random starting point within each cluster 30. Data were collected from 191 respondents between December 2 and December 14, 2016, using Samsung Tab E and Google Nexus 7 tablets, as well as paper surveys, via in-person interviews with one adult member of each selected household. Interviewers were routed to each location with a map generated by Collect SMART.

**Location of Brazos County, Texas.**
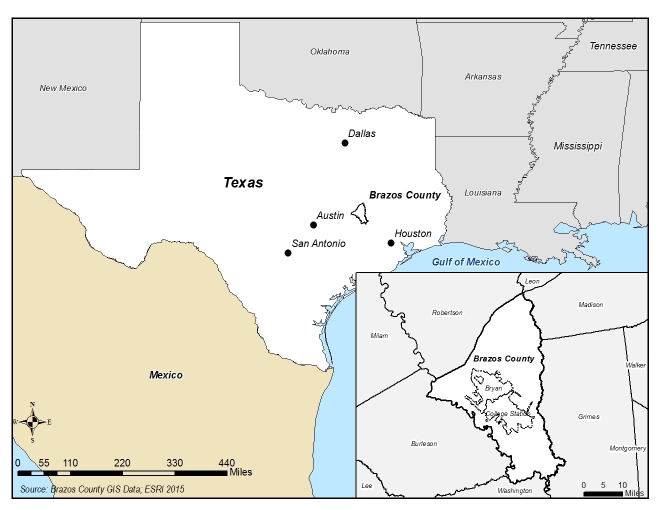
Map created using ESRI ArcMap 10.4. Base layers were adapted from the United States Census Bureau TIGER/Line downloadable shapefiles (https://www.census.gov/geo/maps-data/data/tiger-line.html), and the Brazos Central Appraisal District (http://www.brazoscad.org/gis/).

**Study Variables**

To identify elements that should be included on the CASPER survey to assess the prevalence of risk factors for NTDs, a literature review focusing on each of the diseases in question was performed focusing on the dynamics of NTD transmission in Central and South America 31. Several factors were associated with an increased risk of most or all of the diseases of interest, including immigrant status 32 and rural poverty 2. Unique risk factors for certain NTDs transmitted via insect vectors include a lack of window screens or air conditioning, urban density, standing water near the household, failure to use DEET, and not regularly wearing long sleeves when outside 3. The final survey included 44 questions organized into five sections: demographics, travel history, housing characteristics, vector contact and prevention, and pets and animals 31.

Demographics

Because factors such as overcrowding and poverty are associated with the transmission of NTDs 2^,^^3^, respondents were asked to report the number of people living in their household and whether or not their households’ average income is above or below federal poverty guidelines 33.

Travel History

Although isolated cases of autochthonous transmission of some NTDs has been demonstrated in Texas, travel remains an important risk factor for these diseases. Accordingly, the survey asked respondents to report if anyone in the household has traveled outside the U.S. in the last 3 months, specifically to counties in South and Central America and the Caribbean, as well as the duration of the trip. Survey participants were asked whether they had hosted visitors from other countries in their household, and if so, the country of those visitors’ residence.

Household Characteristics

Housing quality is an important determinant of individual risk, largely because low quality housing often increases interaction between humans and vectors 3. In order to gauge human-vector interaction inside the home, questions about air-conditioning, window screens, cracks or gaps in home structure, and the presence of bugs inside the home were included. Also included in the housing quality section were questions about water usage, food cleanliness, and waste removal services.

Vector Contact and Prevention

When considering vector-borne diseases, it is important to document what actions community members are taking to control vector populations around their households. Therefore, this section of the survey included questions asking about the frequency of bites, the use of prevention behaviors like DEET and long-sleeved clothing, and the number of artificial mosquito breeding habitats, such as tires and flower pots, around their home.

Pets and Animals

Animals can also increase NTD risk in a household, acting as food sources, carriers, and intermediate hosts of infection. Therefore, questions in this section asked about household pets, whether chickens or pigs are raised in or near the house, and about the prevalence of stray dogs near the residence.

**Interviewer Observations**

In addition to directly asking questions to respondents, surveyors asked permission to assess certain aspects of the house, such as the number of visible artificial containers and any noticeable housing quality defects.

**Data Analysis**

A database was created in EpiInfo 7, and data from paper-based surveys were entered into the database. Two 10% samples were double-entered and data collected electronically with tablets were uploaded directly into the EpiInfo 7 database for analysis. The count, percentage, and 95% confidence interval (CI) was calculated for each question using the complex sample frequencies command in EpiInfo. Questions asked of all respondents were weighted to account for the cluster sampling design. Each household received a relative weight based on the number of households in its cluster. Questions asked to a subset of respondents were not weighted.

## Results

A total of 747 homes were approached by an interview team. Of these, contact was made with a resident 308 times, a contact rate of 41.2%. Of those reached, 62%, or 191 total, completed the survey (e.g., cooperation rate), an overall completion rate of 91% (191 of the goal of 210). One hundred and fifteen residents refused to be interviewed, and in 2 cases interviewers encountered a language barrier.

**Demographics**

Consistent with known demographic information about Brazos County, survey respondents were majority white (128 of 191, 69%) or Hispanic (39 of 191, 21.4%). African Americans made up a small portion of the sample (8 of 191, 4.5%) and were under-represented compared to 2010 U.S. Census data. Sample data also overestimated the average number of individuals per household and the individual poverty rate (Table 1).

**Table:** **Table 1:** Demographic characteristics of sampled households in Brazos County, Texas, compared to U.S. Census.

	Sampled Households	95% Confidence Limit	2010 U.S. Census Brazos County
Population size	191	N/A	194,851
People per household	2.9	N/A	2.53
Race/Ethnicity			
Black	4.5%	(4.4-4.7)	10.4%
Hispanic	21.4%	(21.1-21.7)	17.4%
White	69.0%	(68.9-69.3)	65.5%
Other	6.0%	(5.82-6.15)	6.7%
Households in poverty*	25.3%	(24.9-25.6)	27.9%*

*2015 American Community Survey data used for individual poverty estimates

**Travel History**

A total of 12% (23 of 191) of respondents reported travel outside the U.S. in the last three months. Further, just over 5% (10 of 191) of respondents reporting travel to Central or South America, areas that are endemic to the neglected tropical diseases of interest to this study. Some respondents (20 of 191, 10%) reported hosting visitors from foreign countries; specifically, 5.2% (10 of 191) of respondents hosted visitors from Central or South America. Another 10.1% (19 of 191) reported previous residence in a country outside of the U.S., with 3.1% (6 of 191) of respondents indicating residence in a Central or South American country.

**Table:** **Table 2:** Travel characteristics, Brazos County, Texas.

	Percent (%) Prevalence	95% Lower Confidence Limit	95% Upper Confidence Limit
Traveled outside of U.S.	12.2	11.9	12.4
-Endemic area*	5.2	2.5	9.4
-Other area	5.8	2.9	10.1
-Length of visit			
--Less than 7 days	2.1	0.6	5.3
--7 to 14 days	2.1	0.6	5.3
--More than 14 days	4.2	1.8	8.1
--Travel length not given	3.7	1.5	7.4
Hosted recent visitors	10.0	9.7	10.2
-Endemic area	5.2	2.5	9.4
-Other area	5.2	2.5	9.4
Length at current address			
-Less than 6 months	22.4	22.2	22.7
-6 months to 1 year	11.7	11.5	11.9
-More than a year	64.9	64.6	65.2
Previous residence outside U.S.	10.1	9.9	10.3
-Endemic area	3.1	1.2	6.7
-Other area	5.2	2.5	9.4

*Endemic area defined as all Central and South American countries

**Housing Characteristics**

A majority of respondents reported living in houses with air conditioning and window screens, but nearly 30% (55 of 191) of respondents’ housing units contained cracks, gaps, or other holes that may allow insects to access to the home’s interior. About one-third of respondents (61 of 191; 31.9%) reported seeing mosquitoes inside their homes, with 15 of 61 (24.6%) reporting seeing mosquitoes always (8 of 15) or frequently (7 of 15).**** Smaller percentages reported seeing sandflies or kissing bugs (2.1% and 3.7% respectively). Residents reported high confidence in the quality of their water, and waste removal services were provided to a large majority of respondents (158 of 191; 83.1%) by either their city or Brazos County.

**Table:** **Table 3:** Housing characteristics, Brazos County, Texas.

	Percent (%)	95% Lower Confidence Interval	95% Upper Confidence Interval
Households with air conditioning	98.3	98.2	98.4
Window screens on all windows	84.4	84.1	84.6
Cracks, gaps, holes in structure	29.8	29.5	30.1
Seen bugs in home	55.0	54.6	55.3
-Mosquitoes	31.9	25.4	39.1
-Sandflies	2.1	0.6	5.3
-Kissing bugs	3.7	1.5	7.4
Use city water source for:			
-Drinking	61.9	61.6	62.2
-Bathing	93.3	93.1	93.4
-Washing produce	92.8	92.6	93.0
-Washing clothes/dishes	93.7	93.6	93.9
Wash fresh produce regularly	82.9	82.6	83.1
Rated quality of water	4.0 (out of 5.0)	N/A	N/A
Waste removal by city or county	83.1	82.9	83.4

**Vector Contact and Prevention**

A majority of respondents reported being bitten by mosquitoes in or around their home, with only 21.7% (41 of 191) responding that they had never been bitten. Smaller percentages of respondents recall ever being bitten by a sandfly or kissing bug.

**Table:** **Table 4:** Vector contact and prevention, Brazos County, Texas.

	Percent (%) Prevalence	95% Lower Confidence Limit	95% Upper Confidence Limit
Frequency of mosquito bites			
-Always	8.5	8.3	8.7
-Frequently	23.0	22.7	23.3
-Sometimes	45.8	45.4	46.1
-Never	21.7	21.4	22.0
Ever bitten by sandfly	3.7	3.6	3.9
Ever bitten by kissing bug	3.0	2.9	3.1
Wear long sleeved shirts and pants			
-Always	14.1	13.9	14.4
-Frequently	15.0	14.8	15.3
-Sometimes	53.3	52.9	53.6
-Never	16.7	16.4	16.9
Wear DEET mosquito repellent			
-Always	13.5	13.2	13.7
-Frequently	17.6	17.4	17.9
-Sometimes	35.6	35.2	35.9
-Never	31.7	31.4	32.0
Outside around dusk and dawn			
-Always	8.4	8.2	8.6
-Frequently	33.7	33.4	34.1
-Sometimes	37.3	37.0	37.7
-Never	16.1	15.8	16.3

Flower pots were the most frequently reported artificial container by households (68 of 191, 35.8%) followed by pet water dishes (54 of 191, 28.3%) and bird baths (36 of 191, 19%). Six percent (11 of 191) of sampled households reported having old tires on their property, an estimated 4,660 households in Brazos County.

**Table:** **Table 5:** Number of containers per household, Brazos County, Texas.

	Percent (%) Prevalence	95% Lower Confidence Limit	95% Upper Confidence Limit	Mean Containers Per Household	Median Containers Per Household
Bird bath	19.0	18.7	19.3	1.0	1.0
Tires	6.0	5.8	6.2	4.0	3.0
Pet water dishes	28.3	28.0	28.6	3.4	1.0
Flower pots	35.8	35.4	36.1	5.8	3.0
Fountains	6.8	6.6	7.0	Not observed	Not observed
Yard ornaments	11.1	10.9	11.4	Not observed	Not observed
Rain barrels	5.4	5.3	5.6	1.3	1.0
Pools	12.9	12.6	13.1	1.0	1.0

**Pets and Animals**

A majority of respondents reported pet ownership (59.2%, 112 of 191), and households owned an average of 2.4 animals. Nearly four in five (78%, 84 of 112) pet owners reported that their pets slept indoors at night. About 50% (78 of 191) noted the presence of stray dogs within one mile of their home, with 11% (20 of 191) reporting always or frequent sightings. Very few respondents reporting raising chickens or pigs (5% and 1.1%, respectively).

**Table:** **Table 6:** Pets and animals, Brazos County, Texas.

	Percent (%) Prevalence	Mean	Median	95% Lower Confidence Limit	95% Upper Confidence Limit
Have pets	59.2	N/A	N/A	58.9	59.6
-Average number	N/A	2.4	2.0	Standard Deviation: 2.8	
-Sleep indoors	78.5	N/A	N/A	69.5	85.9
Noticed stray dogs (within 1 mile)					
-Always	3.6	N/A	N/A	3.5	3.7
-Frequently	7.4	N/A	N/A	7.2	7.6
-Sometimes	30.1	N/A	N/A	29.7	30.4
-Never	49.2	N/A	N/A	48.8	49.5
Raise chickens	5.0	N/A	N/A	4.8	5.1
Raise pigs	1.1	N/A	N/A	1.1	1.2

## Discussion

To our knowledge, this is the first CASPER to be conducted in Brazos County, Texas. This CASPER was also novel because it was the first CASPER attempted solely for the purpose of assessing the prevalence of risk factors for neglected tropical diseases. For non-vector-borne diseases, we found little exposure to risk factors, with more than 90% of respondents reporting the use of city water for bathing, washing produce, and washing clothes, and 83% reporting using city sewer for waste disposal. Overall, our data suggest that the Bryan/College Station community also has a low prevalence of most risk factors for vector-borne NTDs (e.g., few report seeing or being bitten by sandflies or kissing bugs and most use air conditioning and have window screens), but several factors point to the potential for isolated local transmission or travel associated NTD cases in the future. For example, a majority of residents report sometimes or never wearing long sleeved shirts and pants or using mosquito repellent with DEET, two of the four recommendations made by CDC to prevent mosquito bites 34. This presents public health authorities with clear avenues for implementing health education and intervention campaigns aimed at reducing exposure to risk factors for vector borne NTDs.

Conducting surveillance of potential breeding areas is also important. In this survey, the number of self-reported breeding areas (e.g., tires, pet bowls, and flower pots) was higher than the number observed by the interviewer. One explanation for this may be that there are additional potential breeding sites in backyards, where health department officials and CASPER surveyors do not look. This also presents an opportunity for local public health and other agencies and officials to stress the importance of residents’ implementing outdoor control measures around their homes, such as throwing out, or regularly emptying and cleaning any items that hold water.

International travel is another potential avenue for education and intervention, since 1 in 8 residents of Brazos County indicated travel outside the U.S. in the three months prior to the survey. Local officials should coordinate messaging with federal, state, and transportation authorities to ensure the effectiveness of messages being provided to travelers at airports and other locations. More research is likely needed about the modes of transportation used, and the specific location of travel, particularly since Texas is a border state with Mexico, where some of these diseases are endemic. Since Texas A&M University is a major employer in Brazos County, and many residents are also students, any university-sponsored travel should be accompanied by messages highlighting the potential for being infected by or transmitting an NTD.

Although CASPER is well known for its ability to be used to gather reliable data quickly and at relatively low costs, it has several important limitations. Due to the nature of a household survey, only the prevalence of risk factors for households can be assessed. Risk factor prevalence elsewhere (e.g., public spaces or workplaces) are typically not assessed as part of CASPER. Therefore, the effectiveness of interventions or other programs suggested to reduce the prevalence of risk factors put in place by local governments or public health agencies cannot be assessed at those locations, only at the household level. CASPER surveys collect cross-sectional, self-reported information, which is not verified by the interview team. If certain demographic or socially vulnerable groups are less likely to be included in the survey, there is the potential for response bias. For example, in our study, as in a typical CASPER, houses which were deemed either unapproachable or unsafe were not approached by an interview team; however, residents of these houses may be at greater risk due to poor housing quality or inconsistent removal of water from breeding containers for mosquitoes, leading to potential response bias. Recall bias can also be a concern when asking about travel or food handling behaviors that may have occurred in the past. Since Bryan-College Station has a large percentage of households occupied by students, surveyors could not be sure if respondent’s provided information about their own income or if they were a dependent of their parents, their parents’ income. To address this, income data was calculated at the individual level and compared to the individual poverty rate from the 2010 U.S. Census.

Due to the large immigrant population of the State of Texas and Brazos County, immigration status and personal travel history may potentially be sensitive subjects for respondents. To address this concern, respondents were assured of the confidentially of their responses as part of the informed consent process. Houses that presented with a language barrier (N=2) may also have been more likely to have residents or visitors from foreign countries. To minimize this problem, as many bilingual interviewers as possible were recruited for the survey teams, and teams with a fluent Spanish speaker were assigned to clusters located in the areas of Brazos County where, according to the U.S. Census, more Spanish speakers live. Further, all interviewers were equipped with Spanish copies of the survey in the event that a translator could be found in the household.

One problem we anticipated was the potential for residents to misclassify the presence of mosquitoes, sandflies, and kissing bugs and the prevalence of their bites. It is highly likely that not all respondents knew exactly how to identify these insects or to determine with certainty if they had been bitten by them. To address this issue, surveyors were equipped with 8.5 x 11 color photos of the species of these insects common in Brazos County. When respondents indicated a lack of knowledge, surveyors showed respondents what the insects looked like and explained the details of their bite. For kissing bugs, since many look-alike species exist, to control for misclassification, pictures of these species were shown to respondents. This process gave our team the chance to do educational outreach in the community while collecting data.

## Conclusion

Although NTDs may increasingly pose a threat to health in the U.S., insufficient data exists to ascertain the prevalence and geographic distribution of risk factors relevant to these diseases. Concern over the potential for increasing rates of NTD infection and transmission in Texas led the Texas State Legislature to mandate the establishment of a surveillance program for NTDs. We tested one potential methodology for the surveillance of NTDs; the use of CASPER for assessing the prevalence of risk factors in Brazos County, Texas. Although Texas has a population of more than 25 million, the second largest in the U.S., targeted use of the CASPER method would allow high-risk areas to be identified at the city, county, or regional level. To be effective as a surveillance tool, additional CASPERs should be conducted in areas of the state where there have been more documented cases of NTDs (e.g., the Rio Grande Valley or the City of Houston). After locations with a high prevalence of risk factors are identified, additional public health studies and interventions, such as seroprevalence studies, case interviews and contact tracing, and vector surveillance and sampling could be used to study transmission dynamics in greater detail. Once the prevalence of risk factors for NTDs in Texas is better understood, public health departments can help reduce transmission by making people more aware of the diseases, stressing vector reduction and avoidance of potential hosts like stray dogs, addressing housing quality issues, and encouraging the use of DEET. CASPER is one quick and relatively cost-effective way to learn more about the prevalence of risk factors for NTDs in Texas or elsewhere by identifying a number of avenues for education and prevention by local health departments. The data generated by CASPERs can be immediately actionable, guiding public health priority setting and decision making.

## Data Availability

All data have been deposited publicly at the Texas Data Repository at https://dataverse.tdl.org/dataset.xhtml?persistentId=doi:10.18738/T8/ZHSX6Z (DOI: 10.18738/T8/ZHSX6Z).

## Competing Interests Statement

The authors have declared that no competing interests exist.

## Corresponding Author

Jennifer Horney: horney@sph.tamhsc.edu; 979-436-9391
